# Explainable Artificial Intelligence Paves the Way in Precision Diagnostics and Biomarker Discovery for the Subclass of Diabetic Retinopathy in Type 2 Diabetics

**DOI:** 10.3390/metabo13121204

**Published:** 2023-12-18

**Authors:** Fatma Hilal Yagin, Seyma Yasar, Yasin Gormez, Burak Yagin, Abdulvahap Pinar, Abedalrhman Alkhateeb, Luca Paolo Ardigò

**Affiliations:** 1 Department of Biostatistics and Medical Informatics, Faculty of Medicine, Inonu University, Malatya 44280, Turkey; hilal.yagin@inonu.edu.tr (F.H.Y.); abdulvahappinar7@gmail.com (A.P.); 2 Department of Management Information Systems, Faculty of Economics and Administrative Sciences, Sivas Cumhuriyet University, Sivas 58140, Turkey; yasingormez@cumhuriyet.edu.tr; 3 Computer Science Department, Lakehead University, Thunder Bay, ON P7B 5E1, Canada; aalkhate@lakeheadu.ca; 4 Department of Teacher Education, NLA University College, Linstows Gate 3, 0166 Oslo, Norway; luca.ardigo@nla.no

**Keywords:** type 2 diabetes, diabetic retinopathy, explainable artificial intelligence, biomarkers discovery, diagnostic, Bayesian optimization

## Abstract

Diabetic retinopathy (DR), a common ocular microvascular complication of diabetes, contributes significantly to diabetes-related vision loss. This study addresses the imperative need for early diagnosis of DR and precise treatment strategies based on the explainable artificial intelligence (XAI) framework. The study integrated clinical, biochemical, and metabolomic biomarkers associated with the following classes: non-DR (NDR), non-proliferative diabetic retinopathy (NPDR), and proliferative diabetic retinopathy (PDR) in type 2 diabetes (T2D) patients. To create machine learning (ML) models, 10% of the data was divided into validation sets and 90% into discovery sets. The validation dataset was used for hyperparameter optimization and feature selection stages, while the discovery dataset was used to measure the performance of the models. A 10-fold cross-validation technique was used to evaluate the performance of ML models. Biomarker discovery was performed using minimum redundancy maximum relevance (mRMR), Boruta, and explainable boosting machine (EBM). The predictive proposed framework compares the results of eXtreme Gradient Boosting (XGBoost), natural gradient boosting for probabilistic prediction (NGBoost), and EBM models in determining the DR subclass. The hyperparameters of the models were optimized using Bayesian optimization. Combining EBM feature selection with XGBoost, the optimal model achieved (91.25 ± 1.88) % accuracy, (89.33 ± 1.80) % precision, (91.24 ± 1.67) % recall, (89.37 ± 1.52) % F1-Score, and (97.00 ± 0.25) % the area under the ROC curve (AUROC). According to the EBM explanation, the six most important biomarkers in determining the course of DR were tryptophan (Trp), phosphatidylcholine diacyl C42:2 (PC.aa.C42.2), butyrylcarnitine (C4), tyrosine (Tyr), hexadecanoyl carnitine (C16) and total dimethylarginine (DMA). The identified biomarkers may provide a better understanding of the progression of DR, paving the way for more precise and cost-effective diagnostic and treatment strategies.

## 1. Introduction

Diabetic retinopathy (DR), an ocular microvascular disease, is a common and debilitating complication of diabetes, similar to diabetic neuropathy and nephropathy. DR is the most important etiological factor underlying diabetes-related vision loss [1,2]. The tendency for the onset and progression of this ocular disease is mainly linked to a number of risk determinants, which prominently include long-term diabetes mellitus, hyperglycemia, hyperlipidemia, hypertension, and genetic predispositions [3,4]. Early diagnosis of DR can significantly reduce the disease process and maximize the quality of life and survival time of type 2 diabetes (T2D) patients.

In a systematic nosological classification, DR is divided into non-proliferative diabetic retinopathy (NPDR) and proliferative diabetic retinopathy (PDR) based on the basic criterion of distinguishability of neovascularization. NPDR represents the emerging stage of retinal involvement in diabetes, characterized by the absence of abnormal neovascular formations. PDR marks the peak of retinopathy progression, exemplified by the conspicuous emergence and extensive proliferation of abnormal vessels across the retinal surface [5,6]. Based on the presence or absence of neovascularization, this subclassification system supports the clinical taxonomy of DR. It provides essential guidance for diagnosis, prognostication, and therapeutic interventions in this vision-compromising complication.

Recent strides in metabolomics have revolutionized the quantitative analysis of small molecule metabolites in biological samples, including blood and urine. Understanding the associations between metabolites and biological processes has become paramount, prompting large-scale metabolomics profiling endeavors aimed at unraveling the intricate molecular tapestry of diseases [5,6].

It is essential to highlight that, despite the substantial progress in the field of metabolomics, comprehensive studies focusing on blood metabolites related to DR have been notably limited. Moreover, a number of key metabolites as potential indicators of DR have been identified in some studies in the literature, and their interconnected metabolic pathways have been elucidated, including 2-deoxyribonic acid; 3,4-dihydroxybutyric acid; erythritol; gluconic acid; and ribose [7,8]. These studies underscore the complex relationship between clinical, biochemical, and metabolic biomarkers and the pathogenesis of DR and highlight clear pathways for the development of new diagnostic and therapeutic strategies aimed at addressing this visually debilitating complication.

However, the pathogenesis of DR is complex, and the multitude of contributing factors makes it difficult to identify important biomarkers using traditional only statistical methods due to overfitting and instability. Explainable artificial intelligence (XAI), which has emerged with the loss of trust in the AI model [9,10], is superior in processing high-dimensional data, such as metabolomics, and provides better generalization and differentiation ability, especially in the evaluation of patient health and complications. Using XAI is meant to make it easier to comprehend and diagnose model output, regardless of how accurate the output may be. In conclusion, it will help the user comprehend the results of the system and provide the model’s developer insightful input for bettering the model [11,12]. In one study, the diabetes classification framework based on the XAI method was interpreted and designed by taking into account the results obtained from the Shapley method in the explanations of the model [13]. According to studies conducted in recent years, higher diabetes results were obtained in men with similar body mass indexes (BMI) than in women [14,15]. Since men have more visceral fat than women, men have a higher risk of developing diabetes than women [16,17].

XGBoost has been applied to the diagnosis of chronic kidney disease [18], the classification of cancer patients, and the treatment of epilepsy patients [19]. Specifically, XGBoost has been used to classify atrial fibrillation (AF) and trained a convolutional neural network for electrocardiogram (ECG) annotation. In an effort to classify individual heartbeats, XGBoost was also employed for AF classification [20]. The area under the roc curve (AUC) was used to evaluate the performance of the classifiers on the test set (20%), providing equally stable (sMCI) and progressive (pMCI) local descriptions of four randomly selected test patients, both correctly and incorrectly classified. Explainable boosting machines (EBMs) with and without dual relationships showed high prediction accuracy, with 80.5% and 84.2% accuracy, respectively. In addition, useful clinical insight into how EBM cerebral subdivisions contribute to the diagnosis of Alzheimer’s disease and why a patient is diagnosed with the disease (correctly or incorrectly) is provided [21].

XAI excels at processing high-dimensional data, such as metabolomics, providing better generalization and differentiation capabilities, especially in the assessment of patient health and complications. Although XAI has gained ground in various aspects of diabetes, there is limited research on its application to DR. Therefore, XAI-based research is needed to improve understanding of the complex pathogenesis of DR and potentially improve diagnostic and treatment strategies. Implementing XAI-based models could not only illuminate previously elusive biomarkers but could also significantly enhance diagnostic precision and contribute to more effective, individualized treatment strategies [22,23,24].

Therefore, the present study is conceptualized with the aim of bridging this research gap. Specifically, we intend to employ an XAI-based predictive model to identify candidates for clinical, biochemical, and metabolomic biomarkers across different stages of DR, namely, NDR and NPDR, and among T2D patients. Through this investigation, we seek to contribute a nuanced understanding of the DR pathogenesis landscape and to furnish healthcare practitioners with actionable insights that could facilitate both predictive and preventive care for diabetic patients.

## 2. Materials and Methods

### 2.1. Study Design, Ethical Approval, and Data Features

The current study used a publicly available dataset examining clinical, biochemical, and metabolomic features to explore subclass prediction and biomarkers of DR in T2D patients [25]. The study was conducted according to the principles of the Declaration of Helsinki and was approved by the Inonu University Health Sciences Non-Interventional Clinical Research Ethics Committee (protocol code = 2022/5101). Open-access data on a total of 317 T2D patients (143 NDR patients, 123 NPDR patients, and 51 PDR patients) were used in the study. The diagnosis of DR was made by dilated fundus examination performed by a retina specialist. Gender, age, height, weight, body mass index (BMI), HbA1c, glucose, and creatinine levels of all patients were recorded (Supplementary Materials Table S1). Serum samples were collected from T2D patients with and without DR and stored in a refrigerator at −80 °C in accordance with international ethical guidelines. A targeted metabolomics technique was used to evaluate serum samples from T2D patients. Following quality control processes, 122 metabolites were discovered to identify the DR subclass and were therefore selected for additional statistical studies (Supplementary Materials Table S2).

### 2.2. Classification Algorithms

Artificial intelligence-based medical system diagnoses are frequently used for rapid detection of diseases and risk-free, corrective treatments of detected diseases. As technology evolves, an increasing number of risks and challenges emerge. Medical diagnostic systems are increasingly dependent on artificial intelligence algorithm design. Many studies are being performed in the current environment to provide more appropriate treatment and production in cases that cannot be avoided [26].

In the study, different classification models were created using clinical, biochemical, and metabolomic biomarkers associated with DR in T2D patients. The aim was to obtain a successful prediction model to predict the DR subclass. In this context, three different classification algorithms were used.

eXtreme Gradient Boosting (XGBoost): XGBoost is a high-performance classification algorithm that has been developed by optimizing and enhancing the gradient boosting algorithm through various modifications. This method was initially proposed by Chen and Guestrin, and it has been claimed to work ten times faster than popular classification algorithms. XGBoost, which is based on decision trees, aims to achieve superior results with fewer computational resources [27].

Natural Gradient Boosting for Probabilistic Prediction (NGBoost): NGBoost, proposed by Duan and others, aims to perform predictive uncertainty estimation through gradient boosting with probabilistic predictions, including real-valued outputs. The NGBoost algorithm, developed as open-source software, consists of three components: base learners, distribution, and scoring rule [28].

Explainable Boosting Machine (EBM): EBM is a tree-based, cyclic gradient-boosting generalized additive model that incorporates automatic interaction detection. EBMs have gained recognition for their ability to achieve accuracy levels comparable to state-of-the-art blackbox models, all the while offering complete interpretability. While it is worth noting that EBMs may require more time for training compared to some modern algorithms, they compensate for this by being exceptionally compact and delivering rapid predictions during inference [21,29].

### 2.3. Feature Selection Algorithms

Classification algorithms were combined with feature selection algorithms to determine the importance of biomarkers associated with DR in T2D patients. In this context, minimum redundancy and maximum relevance (mRMR) and Boruta feature selection methods were used. Additionally, due to its inherent ability to calculate the importance of features during training, EBM was employed as a feature extraction algorithm in this study.

Minimum Redundancy and Maximum Relevance (mRMR): The mRMR method, initially proposed by Ding and Peng, aims to select features that are most relevant to class labels by eliminating unnecessary features [30,31]. To achieve this goal, it strives to select features that have minimal correlation with each other. In the first step of the algorithm, the mutual information value is calculated for each pair of features. Using these calculations, minimum redundancy and maximum relevance are determined.

Boruta: Boruta is created using the random forest classifier and aims to iteratively eliminate less relevant features using statistical methods. In the Boruta method, the Random Forest algorithm is run to calculate the Z-score. The highest Z-score among shadow features is identified, and real features with Z-scores higher than this shadow feature are marked. For each feature, statistical tests are then applied using the highest-scoring shadow feature to label the features as either important or unimportant [32,33].

### 2.4. Validation Method and Performance Metrics

In our study, we used a dataset containing three different classes, covering 317 examples with 145 features. To create the dataset ML models, 10% of the data was divided into validation sets and 90% into discovery sets. The validation dataset was used for hyperparameter optimization and feature selection stages, while the discovery dataset was used to measure the performance of the models. A 10-fold cross-validation technique was used to evaluate the performance of ML models. While working on small datasets, the ideal choice is k-fold cross-validation with large k value (but smaller than the number of instances) [34]. Cross-validation is a technique used in machine learning to assess the performance of a predictive model. The 10-fold cross-validation method is a specific type of cross-validation where the dataset is split into 10 subsets or “folds”. The process involves training the model 10 times, each time using a different fold as the test set and the remaining nine folds as the training set. The main advantage of using cross-validation, and specifically 10-fold cross-validation, is that it helps ensure a more reliable evaluation of the model’s generalization performance. It provides a better estimate of how well the model will perform on unseen data compared to a single train–test split [35].

Accuracy: Accuracy can be defined as the ratio of correct predictions to total predictions across all classes or as the rate of correctly categorized data that the machine-learning model that has been trained achieves. In order to explain a particular outcome, statistical modeling often aims to strike a balance between parsimony and accuracy [36].

Precision: This can be expressed as the ratio of the entire quantity of samples that were classified as positive to the amount of genuine positive samples revealed by the classifier. Precision is a helpful metric when minimizing the number of false positives [37].

Recall: When there is an uneven distribution of the data, it is crucial to ascertain the classifier’s sensitivity and specificity values. The classifier’s sensitivity establishes how well it can identify true positives or instances of the event that are actually present in the data under investigation. Stated differently, it represents the likelihood that a set of data that has been identified as belonging to this positive class will continue to be classified as such following the test. For instance, a patient’s test results may suggest that he’s becoming ill even though he does not actually have cancer. It bears the label [38].

F1-Score: The feature selection technique known as the F-score is based on statistics. It evaluates each feature separately in order to sort the pertinent features and it is a measure of truth [39].

AUCROC: One popular metric for assessing how well machine learning classification models perform is AUCROC. An illustration of a classifier’s performance that plots the true positive rate (sensitivity) against the false positive rate (1-specificity) at different threshold settings is called a ROC curve. A higher AUC denotes better performance. The AUC is a single number that summarized the classifier’s overall performance [40].

## 3. Results

**Figure 1:**
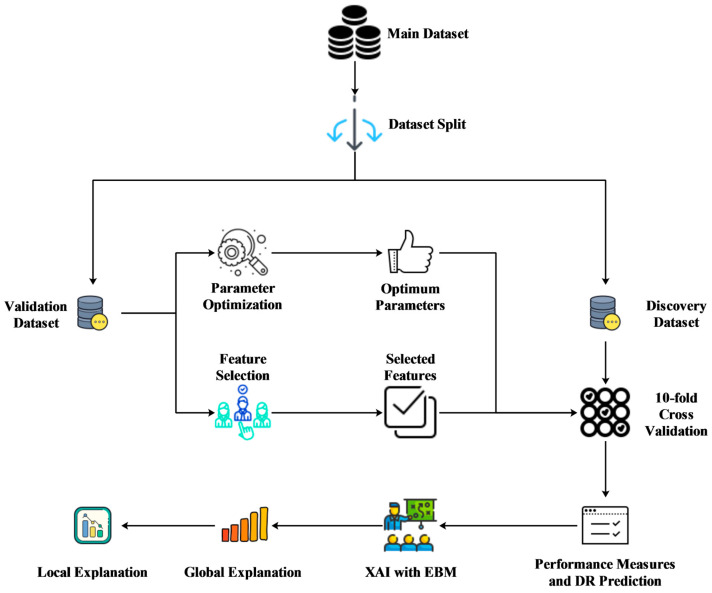
The methodology related to predicting the DR subclass.

The flowchart of the methodology used in the study is presented in Figure 1.

### 3.1. Dataset Preparation

In our study, a dataset containing three different classes, 317 samples, and 145 features was used. Among these, 39 samples had missing values in some features. In the initial stage of our experiment, these missing values were filled by taking the mean values of the respective features. Subsequently, the dataset was divided into discovery and validation datasets. For this purpose, 10% of the samples were randomly selected to create the validation dataset, and the remaining samples were used to form the discovery dataset. The validation dataset will be used for hyper-parameter optimization and feature selection phases, and the discovery dataset will be used to measure performance of models. During the model discovery process and computation of performance metrics, a 10-fold cross-validation technique was employed on the discovery dataset. The purpose of employing a distinct validation set for performance evaluation during hyper-parameter optimization and feature selection processes is to mitigate overfitting. In conclusion, we introduced a separate validation set to effectively address the issue of overfitting [41]. Table 1 shows the number of samples for each class in both the validation and discovery datasets.

### 3.2. Classification Using All Features

In the second stage of the study, classification was performed using all the features in the dataset. Hyper-parameters are crucial factors that affect the performance of classification algorithms. Thus, the hyper-parameters of the XGBoost and NGBoost algorithms, which allow for hyper-parameter configuration, were optimized using the Bayesian optimization method. In pursuit of this objective, the gp_minimize function from the scikit-optimize library was used [42]. Within this function, the acq_func parameter was set to “EI” (Expected Improvement), and the n_calls parameter was chosen to be 50. Table 2 displays the optimized hyper-parameter values for these two methods, along with the highest and lowest values in the hyper-parameter space and the optimum value.

After hyper-parameter optimization, the XGBoost, NGBoost, and EBM methods were trained using 10-fold cross-validation approach on the discovery dataset. In this stage, the XGBoost, NGBoost, and EBM methods were developed using the libraries XGBClassifier, NGBClassifier, and ExplainableBoostingClassifier, respectively [43,44,45,46]. To assess the performance of the trained models’ average values obtained as a result of 10-fold cross-validation for accuracy, precision, recall, F1-Score, and the area under the ROC curve (AUROC) as well as standard deviation (std) between metric scores calculated at each fold, which is shown in Table 3, values were computed.

When the results using all the features presented in Table 3 are examined, it is seen that the most successful model in all performance metrics is EBM, and the second most successful model is XGBoost. Considering the similar results obtained in different performance metrics, it can be interpreted that our model is robust in terms of class types. When analyzing the standard deviation values among folds for each metric, it becomes evident that our model consistently achieves similar results across various situations. This situation led us to conclude that the created model is robust.

### 3.3. Feature Selection

**Figure 2:**
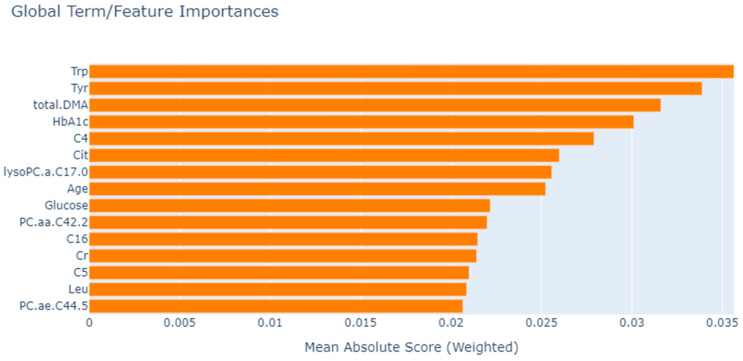
Global biomarker importance of DR in T2D calculated with EBM using all features. Trp: tryptophan; Tyr: tyrosine; total.DMA: total dimethylarginine; HbA1c: glycated hemoglobin; C4: butyrylcarnitine; Cit: citrulline; lysoPC.a.: lysophosphatidylcholine acyl; PC.aa.: phosphatidyl-choline diacyl; C16: hexadecanoyl carnitine; Cr: creatine; C5: valerylcarnitine; Leu: leucine; PC.ae: phosphatidylcholine acyl-alkyl.

After the classification stage, feature selection was conducted to determine the most important biomarkers associated with DR in T2D patients. As mentioned earlier, EBM inherently calculates the importance of biomarkers during training. Figure 2 displays the ranking of importance for the 15 biomarkers calculated using EBM.

In addition to the biomarker importance calculated using EBM, feature selection was also conducted using the mRMR and Boruta methods. In this stage, mRMR and Boruta models were developed using libraries mrmr_selection and BorutaPy, respectively, in the Python language [47,48,49]. The validation dataset was used for feature selection, and the selected biomarker information for each method is presented in Table 4.

After the feature selection stage, models were retrained using the selected biomarkers to observe the difference between using all biomarkers and the selected ones. Models were trained and tested using the discovery dataset with ten-fold cross validation for each classification and the feature selection method, and their performances were calculated using the test dataset. The performance values for each pair are shown in Table 5.

**Figure 3:**
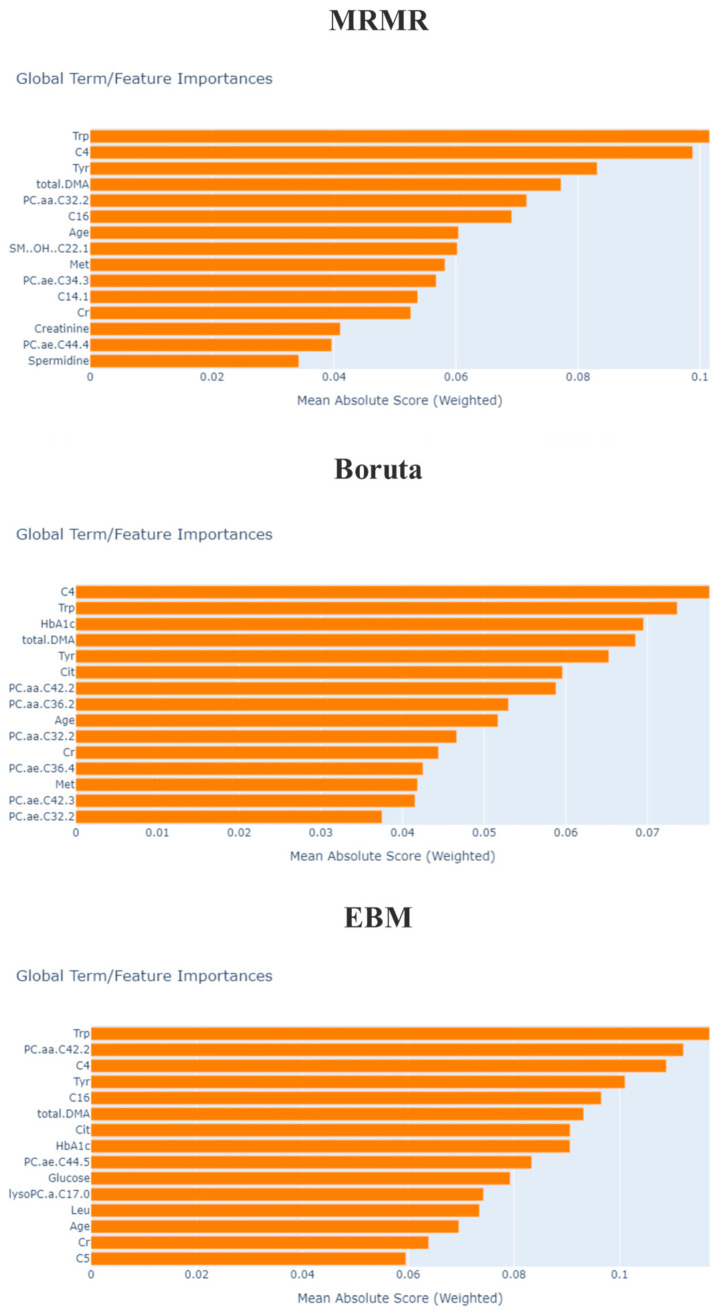
Global biomarker importance of DR in T2D computed using EBM after the feature selection phase. Trp: tryptophan; Tyr: tyrosine; DMA: dimethylarginine; C5: valerylcarnitine; C4: butyrylcarnitine; PC.aa: phosphatidylcholine diacyl; Lys: lysine; Met: methionine; Val: valine; lysoPC.a: lysophosphatidylcholine acyl; C14.1: tetradecenoylcarnitine; PC.ae.: phosphatidylcholine acyl-alkyl; Pro: proline; SM..OH..; hydroxysphingomyelin; C16: hexadecanoyl carnitine; Cr: creatine; Leu: leucine; Cit: citrulline.

Upon examining the results in Table 5, it is observed that the best performance is achieved when EBM is used for feature selection and XGBoost is used as the classification method. When the results in Table 3 and Table 5 are compared, it is evident that determining the importance of biomarkers through feature selection and using only the significant metabolic profiles enhances the success rate in disease type prediction. Therefore, in the design of a biomarker, using only the important biomarker would be sufficient, reducing costs and effort. Another result obtained from the experiment is that the importance order of biomarkers in disease subclass prediction changes after feature selection. To demonstrate this change, after feature selection for each method, the importance ranking of the biomarker is calculated with EBM global explanations, and Figure 3 displays the importance ranking of the selected biomarkers for each method when the EBM model is trained.

EBM is a generalized additivity model based on the tree-based model. The distribution of features can be ranked and plotted to provide the impact on individual prediction from both global and local perspectives due to additivity. The general description of the EBM allows visualizing the consequences of its parameter information on the predicted DR subclass. Since the model achieved the best performance after EBM feature selection, we based the final global explanations of the model on this. As a result, it was observed that tryptophan (Trp), phosphatidylcholine diacyl C42:2 (PC.aa.C42.2), butyrylcarnitine (C4), tyrosine (Tyr), hexadecanoyl carnitine (C16) and total dimethylarginine (DMA) levels played a role as a biomarker candidate in DR subclass prediction.

**Figure 4:**
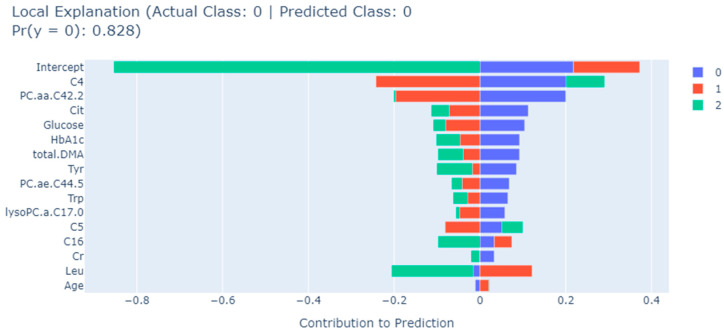
EBM local explanation of the NDR prediction using the XGBoost model. 0: NDR; 1: NPDR; 2: PDR; Trp: tryptophan; Tyr: tyrosine; DMA: dimethylarginine; C5: valerylcarnitine; C4: butyrylcarnitine; PC.aa: phosphatidylcholine diacyl; lysoPC.a: lysophosphatidylcholine acyl; PC.ae.: phosphatidylcholine acyl-alkyl; C16: hexadecanoyl carnitine; Cr: creatine; Leu: leucine; Cit: citrulline.

**Figure 5:**
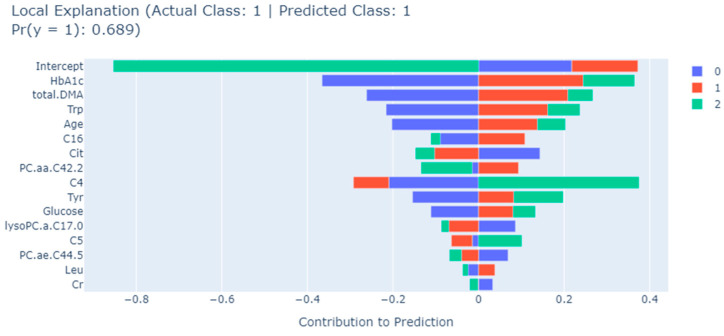
EBM local explanation of the NPDR prediction using the XGBoost model. 0: NDR; 1: NPDR; 2: PDR; Trp: tryptophan; Tyr: tyrosine; DMA: dimethylarginine; C5: valerylcarnitine; C4: butyrylcarnitine; PC.aa: phosphatidylcholine diacyl; lysoPC.a: lysophosphatidylcholine acyl; PC.ae.: phosphatidylcholine acyl-alkyl; C16: hexadecanoylcarnitine; Cr: creatine; Leu: leucine; Cit: citrulline.

**Figure 6:**
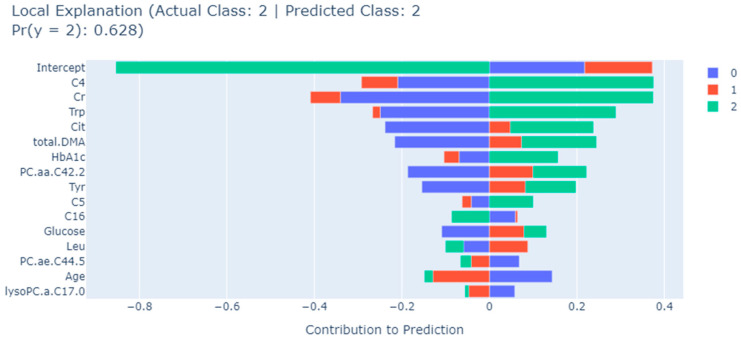
EBM local explanation of the NPDR prediction using the XGBoost model. 0: NDR; 1: NPDR; 2: PDR; Trp: tryptophan; Tyr: tyrosine; DMA: dimethylarginine; C5: valerylcarnitine; C4: butyrylcarnitine; PC.aa: phosphatidylcholine diacyl; lysoPC.a: lysophosphatidylcholine acyl; PC.ae.: phosphatidylcholine acyl-alkyl; C16: hexadecanoyl carnitine; Cr: creatine; Leu: leucine; Cit: citrulline.

The EBM algorithm also allows detailed assessments of contributions of biomarkers to a single prediction. As an example, Figure 4, Figure 5 and Figure 6 show the results of a typical individual prediction for the NDR, NPDR, and PDR subclasses, respectively. In terms of the contribution of each biomarker to the predicted NDR results, the levels of Leu and age biomarkers negatively affected the predicted results, while all other biomarkers had a positive effect (Figure 4). According to Figure 5, in the NPDR prediction results, all biomarkers except Cit, C4, lysoPC.a.C17.0, C5, and PC.ae.C44.5 levels contributed positively to the prediction of the XGBoost model (Figure 5). Moreover, when the EBM explanation regarding the PDR patient was examined, it was determined that the levels of C16, Leu, PC.ae.C44.5, age, and lysoPC.a.C17.0 metabolites contributed negatively to the prediction. In addition, all other biomarkers positively affected the PDR prediction, and the relevant levels of these biomarkers increased the risk of PDR (Figure 6).

## 4. Discussion

The well-known microvascular consequence of diabetes mellitus (DM), DR, is a significant global health issue that places a significant strain on the healthcare system [50]. Since DR is among the leading causes of vision loss globally, accurately predicting its presence is vital for planning, implementing, and evaluating the necessary interventions. Thus, early diagnosis and treatment can help prevent or slow the progression of the condition and reduce the risk of vision loss [51]. Therefore, it is essential to identify clinically useful biomarkers for the early diagnosis and treatment of DR. In this context, metabolomics can provide valuable insights into the metabolic alterations occurring in the retina and the remainder of the body in response to high blood sugar levels and other factors related to diabetes. On the other hand, for this aim, combining metabolomics and machine learning can enhance our understanding of DR, leading to more precise and personalized healthcare strategies [52].

In this study, three different classification algorithms based on metabolomic profile, namely XGBoost, NGBoost, and EBM, were first applied on the original data set to classify the course of DR (NDR, NPDR, and PDR) in T2D patients. Since metabolomics data are generally high dimensional, it poses a great challenge in terms of decision-making in analysis and performance in modeling. Feature selection has proven to be an effective method for dealing with this challenge, both theoretically and in practice [53]. For this reason, three different feature selection methods based on mRMR, Boruta, and EBM were used to identify important metabolites related to DR subclasses and increase the performance of the DR prediction model. All prediction models were then rebuilt using a smaller number of potential target metabolites, and the results were compared. The findings support the information in the literature given that the performance of models created by applying feature selection increases.

Considering all performance metrics of three different classification methods on the original dataset (without feature selection), the accuracy, precision, recall, F1-Score, and AUROC values achieved were 89.51%, 89.45%, 89.51%, 89.48%, and 97.00%, respectively, for EBM. After applying feature selection methods, the best performance in DR prediction was achieved when EBM was used for feature selection and XGBoost was used as the classification method. Therefore, EBM for biomarker discovery in DR and XGBoost algorithms for prediction were identified as the optimal method. After EBM, accuracy, precision, recall, F1-Score, and AUROC values were 91.25%, 89.33%, 91.24%, 89.37%, and 97%, respectively, using XGBoost, which is the optimal model. According to the best-performing EBM feature selection model, the six most important biomarkers that could be used as possible biomarkers in determining the course of DR were Trp, PC.aa.C42.2, C4, Tyr, C16, and totalDMA.

In the literature, there are studies on the classification of DR and the identification of potential biomarkers with ML methods based on metabolomic data. Li et al. [54] proposed a machine learning algorithm using metabolomic and clinical data for early diagnosis of DR and prevention of permanent blindness. Among the machine learning methods (KNN, GNB, LR, DT, RF, XGBoost, NNs, and SVM) generated using clinical and metabolomic data for DM (n = 69), DR (n = 69) and control (n = 69) groups, DT had the best performance (accuracy = 0.933) and was the fastest. In another study, a back propagation (BP) neural network algorithm and hierarchical clustering analysis were used to identify biomarkers that can be used in the classification and early diagnosis of DR [55].

Trp is an essential amino acid and serves as a precursor for various important molecules in the body, including serotonin, melatonin, and kynurenine. Kynurenine is a metabolite of Trp that plays a role in various physiological and pathological processes, including inflammation and immune responses. There is some research suggesting a potential link between kynurenine and DR, a complication of diabetes that affects the eyes [56,57,58,59]. In these studies, it was determined that the Trp concentration decreased depending on the presence of the disease. In the current study, Trp levels were found to decrease between groups, and these decreased levels were found to be significant among the NDR, NPDR, and PDR groups. This is compatible with the information available in the literature. Therefore, the amino acid Trp can be considered as a biomarker in the course of DR.

Phosphatidylcholine (PC) is the predominant phospholipid in circulation and is predominantly associated with high density lipoprotein (HDL) particles. It contributes to the control of circulating lipoprotein levels, particularly very low density lipoprotein (VLDL) [60]. Plasma phosphatidylcholine (PC) concentrations were observed to be modified in obesity, potentially playing a role in the development of obesity-related hepatic steatosis [61]. There are a number of complex relationships between obesity and diabetic retinopathy. Metabolic syndrome consists of a group of metabolic disorders, including insulin resistance, high blood pressure, high triglyceride levels and low HDL cholesterol levels. Metabolic syndrome may increase the risk of diabetic retinopathy [62]. In addition, obesity is associated with increased inflammation (inflammation) and oxidative stress (accumulation of free radicals that damage cells) in the body. These conditions can damage blood vessels in the retina and contribute to the development of diabetic retinopathy [63]. Therefore, the PC.aa.C42.2 metabolite is a strong biologic biomarker for DR.

Lipids are a crucial component of the retina and are crucial to the retina’s functionality. One of the key reasons advancing DR is abnormal lipid metabolism. The effect of acylcarnitine, a lipid metabolism intermediate, on the formation and course of DR has not yet been explained, even if many studies have been conducted on this subject [64,65]. The results of the study conducted by Wang et al. with 1032 T2D patients revealed that the levels of C4, which is a short-chain acylcarnitine, and C16, which is a long-chain acylcarnitine, differed between groups (DR, NDR) [66]. In this study, increasing levels of C4 metabolite showed a statistically significant difference among all groups (*p* < 0.001). On the other hand, although the increased levels of C16 metabolite showed a statistically significant difference both between NDR and NPDR groups and between NDR and PDR groups, the difference between the NPDR and PDR groups was not statistically significant (*p* < 0.001) (Supplementary Materials Table S2). In light of all these results, increased levels of acylcarnitines can be suggested as a biomarker for metabolic abnormalities or a risk factor for DR.

Tyrosine is an amino acid and is important for protein synthesis. This amino acid contributes to various biological functions in the body; in particular, it is involved in the production of thyroid hormones and catecholamines (such as adrenaline, noradrenaline). Diabetic retinopathy refers to damage to the blood vessels in the retina of the eye caused by diabetes. Tyrosine is an important intermediate in the synthesis of catecholamines. Catecholamines are involved in processes, such as stress responses, blood pressure regulation, and energy mobilization. Diabetes can cause metabolic imbalances and stress conditions. In this case, the effect of tyrosine on catecholamine synthesis may increase, and this may increase the pressure on the blood vessels in the retina. On the other hand, oxidative stress and inflammation processes underlie diabetic retinopathy. Tyrosine can contribute to antioxidant systems in scavenging free radicals. However, in diabetes, these antioxidant defense mechanisms may be weakened, leading to increased oxidative stress and damage to blood vessels in the retina. Catecholamines can affect the processes of vasoconstriction (narrowing of blood vessels) or vasodilation (dilation of blood vessels) that act on blood vessels. Endothelial dysfunction in retinal blood vessels plays an important role in diabetic retinopathy. Catecholamines synthesized via tyrosine may act on these endothelial functions and contribute to the deterioration in retinal blood circulation. Finally, diabetes can lead to insulin resistance, and this affects metabolism. Tyrosine is an important precursor for thyroid hormones, and thyroid hormones regulate metabolism. In diabetic retinopathy, factors such as metabolic imbalances and insulin resistance can alter the effects of tyrosine and cause damage to retinal tissue. All this information suggests that tyrosine may be used as a biomarker for DR [67,68].

Total DMA, expressed as the sum of symmetric and asymmetric dimethyl arginine and also suggested as the most important metabolite in determining the course of DR by the EBM model, inhibits the activity of endothelial nitric oxide synthase, an enzyme responsible for the production of nitric oxide. When nitric oxide production is impaired due to elevated levels of dimethyl arginine, there is potential for increased oxidative stress in the blood vessels. Reduced nitric oxide bioavailability can result in an imbalance between the generation of reactive oxygen species and the body’s ability to neutralize them. This imbalance can lead to oxidative stress, which can damage blood vessel walls and contribute to vascular dysfunction. In light of studies in the literature, it can be said that oxidative stress, which is associated with an increase in total DMA, plays an important role in the development of DR [69,70,71,72].

EBM+XGBoost offers the potential for extraction of metabolomic biomarkers in DR subclass prediction. These biomarkers may not only assist clinicians in assessing the severity of DR in a more targeted manner but may also contribute to the optimization of therapeutic interventions. Furthermore, this integrated framework allows monitoring of changes in blood metabolite levels depending on the severity of DR. Such insights can be effective in facilitating early diagnosis and resulting treatment, thereby improving patient outcomes. The ability to track these metabolite changes longitudinally provides an additional layer of analytical depth, allowing healthcare providers to more dynamically tailor treatment regimens based on disease progression or regression.

The achieved results point towards several key implications. First, EBM emerges as a robust method for both classification and feature selection, making it a valuable tool in clinical diagnostics. Second, employing different methods for classification and feature selection could yield superior performance, indicating that a one-size-fits-all approach may not be optimal. Moreover, the improved performance after feature selection validates the importance of this step in model optimization. It could potentially lead to cost-effective tests in medical settings, as only the most relevant biomarkers need to be analyzed. For future research, exploring alternative methods for data imputation during dataset preparation and employing more advanced optimization techniques could be beneficial. Also, further biological validation of the selected biomarkers is needed to confirm their clinical relevance.

## 5. Limitation and Future Works

There are limitations of the current study. External validity, an important concept in ML methods, which is used to evaluate how well a model performs on new datasets other than the one on which it was trained, was not performed using an independent cohort. Therefore, it is recommended that this study should be expanded more comprehensively, and its external validity should be confirmed by including multicenter studies in the future. Furthermore, the models built in this study classify DR based on patients’ demographic, clinical, and metabolomic data. In future studies, patients’ multi-omic (genomic, transcriptomic, proteomic, etc.) information can be included to improve model prediction results.

## 6. Conclusions

In conclusion, the investigative approach that amalgamates XGBoost, a gradient boosting algorithm, with the EBM feature selection technique demonstrates a high degree of efficacy in the accurate prognostication of distinct subclasses of DR. This hybrid methodology harnesses the predictive power of XGBoost while benefiting from the interpretability provided by EBM, thereby achieving a delicate balance between model accuracy and explainability.

## Figures and Tables

**Table 1:** Number of samples for each dataset with respect to classes.

Dataset	Number of NDR Samples	Number of NPDR Samples	Number of PDR Samples	Total Number of Samples
All	143	123	51	317
Discovery	129	111	46	286
Validation	14	12	5	31

**Table 2:** Hyper-parameter space information and optimum hyper-parameter values for the proposed models.

Model	Hyper-Parameter	Hyper-Parameter Space Low Value	Hyper-Parameter Space High Value	Optimum Value
XGBoost	Learning rate	10^−8^	10^−1^	0.02419
	Number of estimator	50	1000	487
	Maximum depth	1	8	5
NGBoost	Number of estimator	50	1000	128
	Learning rate	10^−8^	10^−1^	0.089765

XGBoost: eXtreme gradient boosting; NGBoost: natural gradient boosting for probabilistic prediction.

**Table 3:** Performance values of proposed models calculated using the discovery dataset.

Model	Accuracy (%)	Precision (%)	Recall (%)	FI-Score (%)	AUCROC (%)
XGBoost	86.36 ± 1.91	86.33 ± 1.90	86.36 ± 1.75	86.34 ± 1.84	95 ± 0.19
NGBoost	85.31 ± 1.38	85.86 ± 1.37	85.82 ± 1.27	85.84 ± 1.32	95 ± 0.21
EBM	89.51 ± 1.65	89.45 ± 1.64	89.51 ± 1.83	89.48 ± 1.73	97 ± 0.18

XGBoost: eXtreme gradient boosting; NGBoost: natural gradient boosting for probabilistic prediction; EBM: explainable boosting machine; AUROC: area under the receiver operating characteristic; Performance measures were expressed as mean ± standard deviation.

**Table 4:** Selected biomarker list of DR in T2D computed using each feature selection method.

Model/Algorithm	Selected Biomarker Lists
EBM	Trp, Tyr, total.DMA, HbA1c, C4, Cit, lysoPC.a.C17.0, Age, Glucose, PC.aa.C42.2, C16, Cr, C5, Leu, PC.ae.C44.5
mRMR	Trp, PC.ae.C44.4, Spermidine, C4, C14.1, total.DMA, Tyr, PC.aa.C32.2, Cr, Age, PC.ae.C34.3, Met, C16, SM..OH..C22.1
Boruta	Age, HbA1c, Cr, C4, Cit, Met, Trp, Tyr, Creatinine, total.DMA, PC.aa.C32.2, PC.aa.C34.2, PC.aa.C36.2, PC.aa.C42.2, PC.ae.C32.1, PC.ae.C32.2, PC.ae.C34.2, PC.ae.C34.3, PC.ae.C36.4, PC.ae.C42.3, SM.C24.0

EBM: explainable boosting machine; mRMR: minimum redundancy maximum relevance; Trp: tryptophan; Tyr: tyrosine; DMA: dimethylarginine; Cit: citrulline; C5: valerylcarnitine; C4: butyrylcarnitine; PC.aa.: phosphatidylcholine diacyl; Met: methionine; lysoPC.a.: lysophosphatidylcholine acyl; C14.1: tradecenoylcarnitine; PC.ae.: phosphatidylcholine acyl-alkyl; SM..OH..: hydroxysphingomyelin; C16: hexadecanoyl carnitine; Cr: creatine.

**Table 5:** Performance values of proposed models calculated using the testing dataset after feature selection.

Classification Method	Feature Selection Method	Accuracy (%)	Precision (%)	Recall (%)	FI-Score (%)	AUROC (%)
XGBoost	mRMR	82.16 ± 1.71	82.47 ± 1.61	82.16 ± 1.61	82.32 ± 1.86	89 ± 0.17
	Boruta	87.41 ± 1.29	87.30 ± 1.39	87.40 ± 1.73	87.35 ± 1.84	92 ± 028
	EBM	91.25 ± 1.88	89.33 ± 1.80	91.24 ± 1.67	89.37 ± 1.52	97 ± 0.25
NGBoost	mRMR	81.81 ± 1.22	81.57 ± 1.73	81.80 ± 1.22	81.69 ± 1.49	88 ± 0.29
	Boruta	86.01 ± 1.80	86.18 ± 1.71	86.02 ± 1.23	86.09 ± 1.29	93 ± 0.14
	EBM	88.11 ± 1.41	88.08 ± 1.86	88.10 ± 1.52	88.09 ± 1.21	96 ± 0.25
EBM	mRMR	82.51 ± 1.24	82.41 ± 1.37	82.50 ± 1.57	82.46 ± 1.26	89 ± 0.20
	Boruta	83.91 ± 1.66	83.14 ± 1.29	83.90 ± 1.48	84.51 ± 1.25	90 ± 0.17
	EBM	87.76 ± 1.47	87.72 ± 1.47	87.75 ± 1.62	87.74 ± 1.43	94 ± 0.23

XGBoost: eXtreme gradient boosting; NGBoost: natural gradient boosting for probabilistic prediction; EBM: explainable boosting machine; mRMR: minimum redundancy and maximum relevance; AUROC: area under the receiver operating characteristic; Performance measures were expressed as mean ± standard deviation.

## Data Availability

Data is not publicly available due to “privacy or ethical restrictions”.

## Supplementary Materials

The following supporting information can be downloaded at https://www.mdpi.com/article/10.3390/metabo13121204/s1: Table S1: Statistics on demographic and clinical information; Table S2: Statistics on metabolomics levels.
