# Supplementary material for: Explainable Artificial Intelligence Paves the Way in Precision Diagnostics and Biomarker Discovery for the Subclass of Diabetic Retinopathy in Type 2 Diabetics
Source: Metabolites. 2023 Dec 18;13(12):1204. doi: 10.3390/metabo13121204 (PMC10745306; doi:10.3390/metabo13121204)
Supplement: Supplementary file 1 [file metabolites-13-01204-s001.zip › metabolites-2720044-supplementary.pdf]

Table S1. Statistical results for clinical and biochemical features

Clinical and biochemical feature	DR Subclass**			*p*-value
	NDR	NPDR	PDR	
Age	55a (14)	63b (16.75)	62b (17)	**<0.001**
Height	164 (12)	163 (13)	163 (11)	0.425
Weight	66.5 (13)	65.9 (12)	64 (10.45)	0.640
BMI	23.828 (3.989)	24.49 (3.906)	24.69 (3.926)	0.709
HBA1C	6.8a (1.7)	7.7b (2.8)	8.1b (2.9)	**<0.001**
Glucose	130a (53)	153b (89)	153ab (104)	**0.005**
Creatinine	78.45a (29.15)	81.9a (36.875)	92.9b (72.75)	**<0.001**

*: Features are summarized as 'median (interquartile range)'; #: Kruskal-Wallis H test; **: There is a statistically significant difference in the group categories that do not contain the same letter; NDR: non- diabetic retinopathy; NPDR: non-proliferative diabetic retinopathy; PDR: proliferative diabetic retinopathy.

Table S2. Statistical results for metabolits levels

Metabolites name *	DR Subclass**			*p*-value#
	**NDR**	**NPDR**	**PDR**	
Cr	0.955_{a} (0.237)	1.05_{b} (0.348)	1.145c (0.732)	**<0.001**
C0	44.886 (11.642)	45.153 (12.333)	43.349 (11.806)	0.456
C12	0.09 (0.055)	0.079 (0.043)	0.081 (0.048)	0.056
C14.1	0.1a (0.044)	0.087b (0.04)	0.084b (0.045)	**0.003**
C14.2	0.033 (0.025)	0.031 (0.022)	0.03 (0.026)	0.077
C16	0.123a (0.046)	0.107b (0.037)	0.096b (0.04)	**<0.001**
C18	0.038a (0.014)	0.034b (0.013)	0.03b (0.012)	**<0.001**
C18.1	0.118a (0.054)	0.114b (0.05)	0.103b (0.046)	**0.001**
C18.2	0.079a (0.036)	0.069b (0.03)	0.061b (0.031)	**<0.001**
C2	6.762 (2.618)	6.61 (3.447)	7.805 (4.33)	0.366
C3	0.458a (0.188)	0.484a (0.229)	0.576b (0.318)	**0.002**
C4	0.181a (0.075)	0.198b (0.084)	0.267c (0.148)	**<0.001**
C5	0.115a (0.055)	0.119a (0.053)	0.152b (0.071)	**0.001**
C7.Dc	0.037 (0.025)	0.034 (0.02)	0.035 (0.027)	0.460
C8	0.154 (0.087)	0.143 (0.078)	0.143 (0.099)	0.314
Ala	539.5 (162.25)	549 (150.25)	563 (178.5)	0.238
Arg	108.5 (32.05)	102 (33.075)	110 (29.75)	0.487
Asn	59.6 (13.1)	56.5 (17.7)	58.6 (17.15)	0.125
Asp	19.65 (17.125)	20.05 (16.8)	20.6 (15.95)	0.735
Cit	29.55ab (12.95)	26.95a (16.15)	33.1b (31.05)	**0.018**
Gln	608.5 (125.25)	610 (142.25)	608 (99.5)	0.760
Glu	79.9 (57.525)	88.5 (55.35)	80.4 (54)	0.594
Gly	250 (73.25)	265.5 (100.5)	273 (66.5)	0.070
His	92.45a (14.175)	86.6b (14.775)	87.1ab (16.55)	**0.002**
Ile	98.8 (28.525)	95 (29.95)	98.4 (30.05)	0.714
Leu	194a (44.5)	173b (50.5)	181ab (55)	**0.011**
Lys	226a (45.75)	206.5b (42.5)	207b (48.5)	**<0.001**
Met	25.85a (7.25)	22.9b (6)	23.7b (6.6)	**<0.001**
Orn	83.55 (32.975)	77.9 (39.125)	77.3 (38.25)	0.173

Phe	80.75a (16.2)	72.8b (17.85)	76.4ab (18.75)	**0.017**
Pro	180.5a (72.5)	198b (70.5)	195b (90.5)	**0.018**
Ser	145a (41.25)	139a (40)	131b (40.5)	**0.001**
Thr	144a (36.25)	133.5b (51.5)	133b (56.7)	**0.023**
Trp	64.5a (16.775)	54.35b (17.625)	51.8b (15.65)	**<0.001**
Tyr	72.3a (20.1)	61.9b (20.6)	58.4b (14.4)	**<0.001**
Val	244 (52)	231 (48.5)	235 (54.5)	0.101
Adma	0.515a (0.14)	0.54b (0.139)	0.581b (0.193)	**0.008**
Kynurenine	2.06a (0.852)	2.225ab (0.987)	2.49b (1.41)	**0.004**
Putrescine	0.148 (0.064)	0.147 (0.062)	0.15 (0.072)	0.994
Sarcosine	3.37 (1.19)	3.285 (1.342)	3.54 (1.43)	0.563
Serotonin	0.653 (0.437)	0.653 (0.409)	0.728 (0.515)	0.678
Spermidine	0.244 (0.123)	0.23 (0.117)	0.222 (0.085)	0.064
Taurine	144.5 (65.25)	133.5 (66.5)	132 (52.5)	0.432
Total.Dma	0.794a (0.276)	0.917b (0.399)	1.08c (0.659)	**<0.001**
Lysopc.A.C14.0	3.009 (0.715)	2.827 (0.774)	2.929 (0.814)	0.069
Lysopc.A.C16.0	109.479a (31.835)	96.572b (37.849)	102.163b (27.554)	**<0.001**
Lysopc.A.C16.1	2.947 (0.994)	2.667 (1.516)	2.657 (0.963)	0.083
Lysopc.A.C17.0	1.296 (0.502)	1.409 (0.604)	1.439 (0.643)	0.282
Lysopc.A.C18.0	25.754a (8.908)	22.568b (10.234)	22.413b (7.293)	**<0.001**
Lysopc.A.C18.1	15.242 (4.922)	14.137 (7.259)	13.701 (5.411)	0.058
Lysopc.A.C18.2	25.15a (11.551)	22.288b (10.739)	21.014b (9.948)	**<0.001**
Lysopc.A.C20.3	1.61a (0.78)	1.468ab (0.727)	1.337b (0.599)	**0.011**
Lysopc.A.C20.4	4.703 (2.12)	4.343 (2.225)	4.305 (1.86)	0.194
Pc.Aa.C26.0	0.502 (0.069)	0.506 (0.086)	0.501 (0.088)	0.798
Pc.Aa.C28.1	1.983a (0.656)	1.761b (0.586)	1.8b (0.636)	**<0.001**
Pc.Aa.C30.0	3.031 (1.365)	2.777 (1.44)	2.751 (1.446)	0.088
Pc.Aa.C32.0	13.62a (3.843)	12.645b (4.453)	12.157b (3.906)	**0.003**
Pc.Aa.C32.1	17.695 (13.514)	15.837 (13.444)	16.132 (13.147)	0.125
Pc.Aa.C32.2	3.491a (1.542)	2.786b (1.499)	2.514b (1.818)	**<0.001**
Pc.Aa.C32.3	0.393a (0.159)	0.339b (0.111)	0.358b (0.149)	**<0.001**
Pc.Aa.C34.1	196.364a (79.173)	177.856b (81.117)	181.965ab (72.976)	**0.043**
Pc.Aa.C34.2	356.156a (112.844)	311.615b (96.997)	313.591b (90.195)	**<0.001**
Pc.Aa.C34.3	16.777a (7.506)	14.876b (8.5)	14.744b (6.987)	**0.007**
Pc.Aa.C34.4	1.686a (0.813)	1.396b (0.591)	1.41b (0.619)	**<0.001**

Pc.Aa.C36.0	2.635a (1.178)	2.126b (1.027)	2.035ab (1.485)	**<0.001**
Pc.Aa.C36.1	41.772a (14.027)	35.605b (16.816)	34.425b (10.599)	**<0.001**
Pc.Aa.C36.2	187.317a (58.491)	156.514b (68.897)	161.227b (64.643)	**<0.001**
Pc.Aa.C36.3	104.835a (33.752)	91.268b (34.6)	91.59b (33.826)	**0.002**
Pc.Aa.C36.4	158.82 (56.087)	144.582 (62.087)	147.026 (41.322)	0.072
Pc.Aa.C36.5	44.86 (30.672)	42.908 (24.526)	40.197 (27.18)	0.371
Pc.Aa.C36.6	1.391a (0.703)	1.213b (0.537)	1.323b (0.697)	**0.002**
Pc.Aa.C38.0	3.43a (1.429)	2.799b (1.186)	2.747b (1.58)	**<0.001**
Pc.Aa.C38.1	1.168 (0.592)	0.979 (0.62)	1.084 (0.53)	0.111
Pc.Aa.C38.3	42.578a (14.038)	38.427b (13.163)	37.55b (12.193)	**<0.001**
Pc.Aa.C38.4	83.246 (31.618)	81.445 (32.231)	78.147 (20.492)	0.362
Pc.Aa.C38.5	63.574 (23.443)	62.684 (21.827)	61.54 (22.971)	0.124
Pc.Aa.C38.6	134.927a (56.142)	112.334b (42.674)	112.454b (51.515)	**<0.001**
Pc.Aa.C40.1	0.432 (0.165)	0.408 (0.152)	0.392 (0.167)	0.160
Pc.Aa.C40.2	0.332 (0.13)	0.306 (0.146)	0.283 (0.126)	0.078
Pc.Aa.C40.3	0.561 (0.225)	0.543 (0.224)	0.529 (0.202)	0.230
Pc.Aa.C40.4	2.812 (0.946)	2.803 (1.388)	2.754 (1.214)	0.182
Pc.Aa.C40.5	13.878a (7.379)	12.473b (7.959)	11.732b (5.094)	**0.015**
Pc.Aa.C40.6	49.499a (20.844)	42.339b (17.201)	42.123b (18.191)	**<0.001**
Pc.Aa.C42.0	0.686 (0.327)	0.634 (0.286)	0.649 (0.287)	0.169
Pc.Aa.C42.1	0.407a (0.187)	0.361b (0.132)	0.376ab (0.173)	**0.006**
Pc.Aa.C42.2	0.43a (0.168)	0.36b (0.136)	0.316b (0.189)	**<0.001**
Pc.Aa.C42.4	0.179 (0.062)	0.17 (0.061)	0.169 (0.045)	**0.071**
Pc.Aa.C42.5	0.475a (0.22)	0.442a (0.189)	0.371b (0.178)	**0.002**
Pc.Aa.C42.6	0.745a (0.266)	0.657b (0.251)	0.691ab (0.274)	**0.035**
Pc.Ae.C30.0	0.217 (0.092)	0.206 (0.07)	0.216 (0.097)	0.216
Pc.Ae.C32.1	2.345a (0.698)	1.973b (0.686)	1.985b (0.719)	**<0.001**
Pc.Ae.C32.2	0.602a (0.247)	0.498b (0.181)	0.502b (0.234)	**<0.001**
Pc.Ae.C34.0	1.07a (0.392)	0.975b (0.349)	0.984ab (0.486)	**0.012**
Pc.Ae.C34.1	6.54a (1.58)	6.184b (1.764)	5.82b (2.063)	**0.006**
Pc.Ae.C34.2	7.989a (2.906)	6.245b (2.366)	6.355b (2.718)	**<0.001**
Pc.Ae.C34.3	6.02a (2.218)	5.002b (2.052)	5.089b (2.582)	**<0.001**
Pc.Ae.C36.0	0.945 (0.476)	0.893 (0.365)	0.898 (0.427)	0.059
Pc.Ae.C36.1	5.465a (1.526)	5.183b (1.704)	5.352ab (1.875)	**0.031**
Pc.Ae.C36.2	9.217a (3.849)	8.018b (3.427)	8.719ab (3.237)	**0.004**

Pc.Ae.C36.3	5.315a (1.78)	4.341b (1.604)	4.721b (1.754)	**<0.001**
Pc.Ae.C36.4	11.731a (3.799)	10.148b (4.011)	10.268b (3.676)	**<0.001**
Pc.Ae.C36.5	9.681a (3.588)	8.462b (3.018)	8.405b (3.13)	**<0.001**
Pc.Ae.C38.0	2.566a (1.167)	2.344b (0.797)	2.346b (1.027)	**0.009**
Pc.Ae.C38.1	0.709a (0.332)	0.6b (0.308)	0.647ab (0.384)	**0.003**
Pc.Ae.C38.2	1.609a (0.574)	1.408b (0.596)	1.376b (0.648)	**<0.001**
Pc.Ae.C38.3	2.716a (0.896)	2.429b (0.84)	2.63ab (0.861)	**0.046**
Pc.Ae.C38.4	7.883 (2.258)	7.19 (2.868)	7.58 (2.403)	0.206
Pc.Ae.C38.5	12.97a (4.045)	11.236b (3.71)	10.78b (3.51)	**<0.001**
Pc.Ae.C38.6	7.707a (3.236)	6.549b (2.347)	6.41b (3.099)	**<0.001**
Pc.Ae.C40.1	1.354a (0.533)	1.116b (0.365)	1.147b (0.522)	**0.001**
Pc.Ae.C40.2	1.484 (0.473)	1.335 (0.461)	1.42 (0.497)	0.054
Pc.Ae.C40.3	0.828a (0.211)	0.756b (0.21)	0.781ab (0.237)	**0.020**
Pc.Ae.C40.4	1.721a (0.525)	1.57b (0.562)	1.571b (0.509)	**0.001**
Pc.Ae.C40.5	3.228a (1.193)	2.87b (0.928)	2.816b (1.189)	**<0.001**
Pc.Ae.C40.6	4.933a (1.869)	4.526b (1.73)	4.235ab (1.983)	**0.025**
Pc.Ae.C42.1	0.308 (0.09)	0.296 (0.103)	0.31 (0.096)	0.167
Pc.Ae.C42.2	0.626a (0.171)	0.548b (0.175)	0.545b (0.269)	**<0.001**
Pc.Ae.C42.3	0.839a (0.267)	0.676b (0.27)	0.667b (0.23)	**<0.001**
Pc.Ae.C42.4	0.691a (0.238)	0.599b (0.264)	0.625ab (0.221)	**0.008**
Pc.Ae.C42.5	1.689a (0.541)	1.5b (0.482)	1.509b (0.463)	**<0.001**
Pc.Ae.C44.3	0.119 (0.042)	0.111 (0.032)	0.113 (0.052)	0.101
Pc.Ae.C44.4	0.34 (0.1)	0.305 (0.118)	0.319 (0.082)	0.101
Pc.Ae.C44.5	1.393a (0.598)	1.256b (0.638)	1.296b (0.491)	**0.002**
Pc.Ae.C44.6	1.373 (0.554)	1.3 (0.509)	1.307 (0.458)	0.196
Sm..Oh..C14.1	3.986 (1.185)	3.784 (1.316)	3.906 (1.32)	0.221
Sm..Oh..C16.1	2.449 (0.721)	2.383 (0.873)	2.46 (0.75)	0.493
Sm..Oh..C22.1	10.909a (3.106)	9.415b (3.425)	9.878b (2.474)	**<0.001**
Sm..Oh..C22.2	7.75 (2.391)	7.348 (2.55)	7.45 (1.611)	0.328
Sm..Oh..C24.1	1.15a (0.4)	1.042b (0.437)	1.095ab (0.369)	**0.014**
Sm.C16.0	112.041 (23.037)	104.721 (29.652)	107.116 (21.5)	0.134
Sm.C16.1	15.332 (3.613)	14.978 (5.552)	15.234 (3.463)	0.134
Sm.C18.0	20.77a (6.014)	18.464b (5.525)	18.9b (3.345)	**0.002**
Sm.C18.1	9.453 (2.825)	9.214 (3.259)	9.169 (2.59)	0.253
Sm.C24.0	22.084a (6.556)	19.226b (5.428)	19.22b (5.918)	**<0.001**

Sm.C24.1	45.177 (11.802)	44.458 (13.729)	44.129 (9.832)	0.351
Sm.C26.1	0.394 (0.186)	0.39 (0.188)	0.391 (0.175)	0.806
H1	6554.552a (2667.197)	7453.156b (3610.018)	7762.709ab (4574.336)	**0.035**

*: Features are summarized as 'median (interquartile range)'; #: Kruskal-Wallis H test; **: There is a statistically significant difference in the group categories that do not contain the same letter; NDR: non- diabetic retinopathy; NPDR: non-proliferative diabetic retinopathy; PDR: proliferative diabetic retinopathy.
